# Strengthening breast cancer screening program through health education of women and capacity building of primary healthcare providers

**DOI:** 10.3389/fpubh.2023.1276853

**Published:** 2023-11-16

**Authors:** Ramesh Kumar Sangwan, Ramesh Kumar Huda, Ansuman Panigrahi, G. S. Toteja, Arun Kumar Sharma, Mahendra Thakor, Pankaj Kumar

**Affiliations:** ^1^ICMR-National Institute for Implementation Research on Non-Communicable Diseases, Jodhpur, Rajasthan, India; ^2^ICMR-Regional Medical Research Center, Bhubaneswar, Odisha, India; ^3^Chief Executive Officer, Jodhpur City Knowledge & Innovation Foundation, IIT, Jodhpur, Rajasthan, India; ^4^Department of Community Medicine, University College of Medical Sciences, New Delhi, India

**Keywords:** breast cancer, early detection, breast self-examination, implementation, suspect case

## Abstract

**Background:**

Globally and in India, breast cancer is a prevalent malignancy. India saw 178,361 new cases and 90,000 deaths in 2020. Timely detection is vital, highlighting the importance of Breast Self-Examination (BSE), especially in low-income settings. Strengthening BSE in awareness and screening efforts is urgent. Despite awareness, practical application lags due to women’s reluctance. Effective execution demands partnerships, a multi-sectoral strategy, and training grassroots workers.

**Objective:**

To address these challenges, the present study aims to strengthen the breast cancer screening program using BSE strategy and adopting a referral mechanism for the diagnosis and treatment of suspect cases.

**Methods:**

A community-based study occurred in specific districts of Rajasthan (2017–2022), enhancing breast cancer screening for women aged 30–65. It involved healthcare providers and local women, utilizing tools like the MT-DM-GP6620 Breast Inspection Model, educational booklets, and semi-structured schedules. The strategy encompassed knowledge assessment, capacity building for healthcare providers, BSE training, increasing women’s breast cancer awareness, suspect case referrals, and phone-based follow-up.

**Results:**

Our study encompassed 157,225 women aged 30–65 in Jodhpur, Jalore, and Pali districts. Initial breast self-examination (BSE) awareness was below 1%. BSE training reached 218,978 women using booklets and demonstrations, with 72% aged 30–65 and the rest 15–30. Follow-ups reinforced BSE, leading to 745 identified suspect breast cancer cases, mostly due to painless lumps (332 cases). Capacity-building workshops involving 824 medical and paramedical staff strengthened early breast cancer detection in Jodhpur and Jalore, in collaboration with the district health department.

**Conclusion:**

The study model’s success suggests its applicability in other Rajasthan districts, Indian states, and global breast cancer prevention programs. While positive outcomes were evident, challenges related to culture, cost, and benefits warrant consideration. The approach prioritized early detection through community engagement, reducing patient and government burdens. Community involvement and healthcare engagement were pivotal, with breast self-examination proving effective for enhancing awareness and early detection. Promoting BSE education can significantly enhance breast cancer awareness and early detection.

## Introduction

Breast cancer (BC) is the most common malignancy among women globally. Breast cancer accounts for 14% of cancers in Indian women, being the most prevalent kind (1). In India, 1,62,468 new cases of breast cancer were reported in 2018, along with 87,090 deaths (1). About 54% of the Indian women reported breast cancer at a late stage and the five-year overall survival rate of breast cancer in India ranged from 40 to 62% (2, 3).

The 2020 Globocan study delved into 35 different types of cancer in India, spanning all age groups and genders. The findings revealed an approximate of 1.32 million new cancer cases and 851,000 cancer-related deaths. Within this data, there were 178,361 newly diagnosed breast cancer cases, tragically resulting in 90,000 reported fatalities (4).

The National Cancer Registry Programme Report (NCRP) for 2020 presented data on cancer incidence obtained from 28 Population-Based Cancer Registries (PBCRs) spanning the years 2012–2016. These figures served as the foundation for calculating cancer estimates in India. In the case of females, the total number of all-site cancers is expected to rise to 806,218 in 2025, compared to 627,202 in 2015. Specifically, it is projected that breast cancer cases will top the list, with an estimated 232,832 cases among females in the year 2025.

Early identification and prompt treatment are the most effective interventions for BC management, according to the World Cancer Report 2020 (5). Because breast cancer is frequently discovered in advanced stages in underdeveloped nations, attempts to identify it early may help to shorten the time between diagnosis and treatment, increasing the likelihood of survival and curing the disease as well as making it easier and more affordable to treat (6).In low-income nations compared to high-income countries, early detection of breast cancer by breast self-examination (BSE) is crucial for improving breast cancer outcomes and survival (7).There is an urgent need for interventions to implement and strengthen BSE in the current cancer awareness and screening programs, given the significant role that BSE may play in low-resource settings (8).

Despite the fact that people are aware of breast cancer, its early diagnosis, and screening technologies, and they have a favorable attitude toward them, there is still a big gap that has to be addressed in terms of its application (9). Despite knowing the technique of performing BSE, women do not have a positive attitude toward it and were reluctant to practice BSE (10). For its effective implementation, collaborations with ministries, multi-sectoral approach, strengthening of referral system along with involvement/training of grass root level workers are needed (11). However, most of the female health workers (FHWs) have inadequate knowledge about breast examination skills (12).

The NPCDCS program in India, launched in 2010, aimed to screen for common non-communicable diseases like breast, oral, and cervical cancer in 100 districts. ASHA workers led the screening, with support from ANMs and LHVs.

The Government of Rajasthan focused on preventing and controlling cancer, diabetes, cardiovascular diseases, and stroke. The modus operandi was to organize camps once a month at a fixed location, usually a Community Health Center (CHC), on a specific day. People with suspected diseases, identified by ANMs, were mobilized by them to attend these camps. They were screened for possible NCDs, treated, and referred to the District Hospital (DH) as needed. The training of Medical Officers, LHVs, ANMs, AWWs, ASHA/ASHA Sahyogini, and home visits by AWWs/ASHA had not yet been realized.

With this background, the present study was undertaken with the aim of strengthening breast cancer screening program using BSE strategy and adopting a referral mechanism for the diagnosis and treatment of suspect cases.

## Methods

### Study design and study area

A community-based study was conducted in the selected blocks of Jodhpur, Jalore, and Pali districts of Rajasthan state. There were 10 blocks in Jodhpur, 8 blocks in Jalore, and 11 blocks in Pali, from which 40% of the blocks were randomly selected from each district. Thus, 4 blocks from Jodhpur, 3 blocks from Jalore, and 4 blocks from Pali were included. Specifically, the selected blocks were Bawari, Luni, Mandore, and Bilara in Jodhpur district; Ahore, Jalore, and Sayla in Jalore district; and Jaitaram, Pali, Sojat, and Rohat in Pali district.

### Study population and selection criteria

All the women aged 30–65 years residing in the selected blocks were considered eligible for the study. Women with a terminal illness or those who were non-cooperative were not included in the study. Also, health care providers such as Medical Officers (MOs), Auxiliary Nurse Midwives (ANMs), Lady Health Visitors (LHVs), Accredited Social Health Activists (ASHAs), Anganwadi Workers (AWWs) of the selected blocks were included in the study. Those women not available at home during the time of visit and health care staff not present during the capacity building workshop were excluded from the study. Written informed consent was obtained from the study participants before initiation of the study.

Timelines: The study was conducted from 2017 to 2022. During this period, the following steps were followed:

The first phone call follow-up was conducted after at least one month of BSE training for women. The second phone call follow-up was carried out two months after the first phone call follow-up. Suspect and confirmed cases were also monitored through phone call follow-ups.

Steps 1 to 3 were taken parallel throughout the study period.

### Data collection tools

The following tools were used for the study: (1) A semi-structured pretested schedule to collect information on socio-demographic details, knowledge on risk factors, signs and symptoms of BC, family history of breast cancer etc., (2) Suspect case record form and (3) Phone call follow up form for BSE compliance.

### Interventions

The following tools were used for the interventions: (1) MT-DM-GP6620 Breast Inspection Model, manufactured By the Micro technologies, (2) Education booklet about BC, (3) video.

The model had realistic appearance and precise anatomical structure for inspection and palpation. This natural life size model having implanted innocent tumor and malignant tumor, allowed to practice diagnosis according to the position, size, texture, and movement. Education booklet of breast cancer had pictorial presentation of the symptoms of BC and steps to perform BSE.

During the capacity-building workshop for healthcare professionals, we utilized a 12-min video (by Dr. Reddy Pharmaceuticals) to raise awareness about various aspects of breast cancer, followed by presentations from experts.

We used a breast inspection dummy model and a booklet to generate awareness about breast cancer symptoms, risk factors and breast self-examination (BSE) during house-to-house visits for the community women. Following this, the female research team demonstrated the steps for BSE.

The Breast Inspection Model was very useful for raising awareness about the signs and symptoms of breast cancer and for teaching breast self-examination, especially among illiterate women, but also for healthcare professionals and women in general. We had also used phone call follow up to increase adaptation of BSE practices.

### Study strategy

The study employed Breast Self-Examination (BSE) as its primary strategy, consisting of four components aimed at strengthening breast cancer screening programs in the selected blocks. To achieve this, we reached out to various stakeholders, including community representatives, community volunteers, medical officers, paramedical staff from concerned Primary Health Centers (PHCs) and Community Health Centers (CHCs) in three districts, as well as all block chief medical officers within these selected blocks. We also sought the support of district chief medical health officers and medical colleges. To reach more study participants, we sought the assistance of ASHA, Anganwadi helpers and other volunteers in the community to spread the information the day before the assessment and BSE training. The women in the community voluntarily provided space in their homes for training on BSE.

#### Component I: knowledge assessment and capacity building of health care providers

Using a semi- structured schedule, knowledge of health care providers {(Medical Officers (MOs), Auxiliary Nurse Midwives (ANMs), Lady Health Visitors (LHVs), Accredited Social Health Activists (ASHAs), Anganwadi Workers (AWWs)} was assessed. A three-tier training program was adopted: a) training of the trainers, which includes training of MOs of all the PHCs and CHCs; b) training of all ANMs and LHVs by the trainers (MOs); c) training of all ASHAs/ASHA Sahyogini and AWWs by ANMs and LHVs. First, subject experts imparted training to the medical officers for clinical breast examination to identify suspect cases with low false positivity and false negativity. Then, LHVs and ANMs were trained by the trained MOs regarding clinical breast examination and education of women about the signs and symptoms of breast cancer. LHVs/ANMs in turn trained ASHA/ASHA Sahyogini, AWWs, and proactive local volunteers.

#### Component II: knowledge assessment, BSE training, and breast cancer awareness among women

Using a semi-structured schedule, relevant information regarding demographic details, knowledge about risk factors, signs and symptoms of breast cancer, and family history of breast cancer were collected from the study participants by the project staff. After knowledge assessment house-to-house or group meetings (if feasible) were done by the project staff to educate women about symptoms of BC and BSE. BSE training of women was done using the MT-DM- GP6620 breast inspection model and breast cancer awareness through an educational booklet. Women were asked to report breast cancer symptoms immediately as and when detected by themselves.

#### Component III: identification and referral of suspect cases of breast cancer

A woman reporting any of the following symptoms such as painless Lump in the breast, change in size and shape, swelling in the armpit or collarbone, painful lump in armpit, redness or rash in the breast or nipple, breast skin puckering or dimpling, nipple Retraction, unusual nipple discharge, painful lump in breast, and painless lump in armpit for more than one month was considered as a suspect in the study.

After identification of suspect cases, we record the details of each case, and the project staff was directed to inform the concerned ASHA/ANM/MO regarding the case and motivate the suspect cases to visit nearby PHC/CHC for a clinical breast examination. Those who were suspected to have breast cancer through the clinical breast examination were referred to nearby tertiary health care settings along with ASHA for further investigation and treatment.

#### Component IV: follow up through phone call

A telephone cell was created in the institute and phone calls were made to talk with women participants to know about the compliance of breast self- examination as well as follow-up of the suspect and confirmed cases of breast cancer. Suspect cases were followed up through phone calls/home visits by the project staff/ASHA/ANM and motivated to consult the doctor if any symptoms persisted.

### Data analysis

IBM SPSS Software 21.0 (SPSS Inc. Chicago, IL, United States) was used to clean, edit, and analyse the collected data. Descriptive analysis was employed to describe the data.

### Ethical considerations

Ethical clearance was obtained from the Institute Ethics Committee of ICMR-NIIRNCD. The entire procedure of this study was conducted in accordance with the ethical standards. Participants were given the assurance that taking part in the study was completely voluntary and that they had the freedom to leave at any moment. Prior to performing the survey, each participant had to sign a participant consent form. Data were stored in a confidential, anonymous manner.

## Results

In this study, interventional BSE strategy was adopted to strengthen breast cancer screening program in three districts (Jodhpur, Jalore, Pali) of Rajasthan. Table 1 shows the socio-demographic characteristics of the study population. The majority of women (64,040, 40.4%) belonged to the 30–39-year age category and nearly 90% of the women were married. More than three-quarters of women (118,973, 75.2%) were illiterate and 109,202 (69%) women were housewives. Among 157,225 women, 18,380 (11.6%) were currently consuming tobacco and 224 (0.1%) were consuming alcohol. While enquired about family history (maternal home) of cancer, 567 (0.4%) reported a history of breast cancer in their families whereas 3,743 (2.4%) informed about history of other cancers such as oral cancer, throat cancer, lung cancer, stomach cancer, blood cancer, cervical cancer, etc. in the family. Table 2 depicts the knowledge of women about breast cancer. More than half of the women respondents (89,669, 56.7%) had heard about breast cancer. Almost all the women had either no knowledge or poor knowledge regarding symptoms/risk factors of breast cancer. Very few women (805, 0.5%) reported that they had heard about the role of breast self-examination for early detection of breast cancer and only 321 (0.2%) ever practiced breast self-examination. Regarding breastfeeding practices, nearly 97% of women breastfed their babies.

**Table 1:** Socio-demographic characteristics of women (30–65 years) respondents (*n* = 157,225).

Characteristic	Jodhpur	District Jalore	Pali
	(*n* = 83,478)	(*n* = 34,765)	(*n* = 38,982)
Age group (in years)			
30–39	37,225 (44.6)	12,285 (35.3)	14,530 (37.3)
40–49	18,864 (22.6)	8,454 (24.3)	9,771 (25.1)
50–59	14,732 (17.6)	10,127 (29.1)	8,184 (21.0)
> = 60	12,521 (15.0)	6,080 (17.5)	6,447 (16.5)
Missing	136 (0.2)	2,181 (6.3)	50 (0.1)
Marital status			
Married	76,646 (91.8)	31,261 (89.9)	35,431 (90.9)
U/W/D ^*^	6,815 (8.2)	3,492 (10.0)	3,550 (9.1)
Missing	17	12	01
Education			
Illiterate	61,392 (73.5)	28,787 (82.8)	28,794 (73.9)
Literate	21,473 (25.7)	5,876 (16.9)	10,038 (25.7)
Missing	613 (0.7)	102 (0.3)	150 (0.4)
Occupation			
House wife	61,012 (73.1)	22,839 (65.7)	25,351 (65.0)
Others ^*^	21,309 (25.5)	11,677 (33.6)	13,120 (33.7)
Missing	1,157 (1.4)	249 (0.7)	511 (1.3)
Current tobacco consumption			
Yes	8,401 (10.1)	3,230 (9.3)	6,749 (17.3)
No	74,953 (89.8)	31,507 (90.6)	32,217 (82.6)
Missing	124 (0.1)	28 (0.1)	16 (0.1)
Current alcohol consumption			
Yes	124 (0.1)	50 (0.1)	50 (0.1)
No	83,151 (99.6)	34,693 (99.8)	38,963 (99.9)
Missing	203 (0.2)	22 (0.1)	969 (24.2)
History of cancer in family			
Breast cancer	289 (0.3)	101 (0.3)	177 (0.5)
Others^**^	1726 (2.1)	814 (2.3)	1,203 (3.1)
None	81,463 (97.6)	29,473 (84.8)	37,597 (96.4)
Missing	0	4,377 (12.6)	05

* U: Unmarried, W: Widow, D: Divorced; Others include laborer, farmer, business, service (govt/pvt).

** Others include oral cancer, throat cancer, lung cancer, stomach cancer, cervical cancer, blood cancer, etc.

**Table 2:** Knowledge about breast cancer among women (30–65 years) respondents (*n* = 157,225).

	Jodhpur	District Jalore	Pali
	*N* = 83,478	*N* = 34,765	*N* = 38,982
	*n* (%)	*n* (%)	*n* (%)
If heard about breast cancer?			
Yes	53,660 (64.3)	14,314 (41.2)	21,695 (55.6)
No	29,818 (35.7)	20,451 (58.8)	17,287 (44.4)
Knowledge about symptoms of breast cancer			
None	31,497 (37.7)	21,446 (61.7)	18,829 (48.3)
Poor (1–3)	51,601 (61.8)	13,237 (38.1)	20,067 (51.5)
Middle/High (≥ 4)	380 (0.5)	82 (0.2)	86 (0.2)
Knowledge about risk factor(s) for breast cancer			
None	77,077 (92.3)	32,382 (93.1)	37,255 (95.5)
Poor (1–3)	6,199 (7.4)	2,354 (6.8)	1706 (4.4)
Middle/High (≥ 4)	202 (0.2)	29 (0.1)	21 (0.1)
If heard about breast cancer, do you know about BSE for early detection of breast cancer			
Yes	449 (0.5)	176 (0.5)	180 (0.5)
No	52312 (62.7)	13620 (39.2)	21042 (54.0)
No response	725 (0.8)	518 (1.5)	403 (1.0)
Missing	174 (0.2)	70 (0.2)	0
Source of information for early detection of breast Cancer			
Friends	9 (0.01)	10 (0.029)	
Relatives	18 (0.02)	25 (0.072)	8 (0.02)
Neighbors	13 (0.02)	11 (0.032)	12 (0.03)
Health care provider	84 (0.10)	37 (0.11)	36 (0.09)
Mass-media	36 (0.04)	25 (0.072)	33 (0.08)
Any others	59 (0.07)	70 (0.20)	33 (0.08)
No response	247 (0.30)	5 (0.014)	72 (0.18)
Ever practiced breast self-examination			
Yes	168 (0.2)	85 (0.2)	68 (0.2)
No	63 (0.1)	59 (0.2)	33 (0.1)
Not applicable Missing	218 (0.3)	32 (0.1)	79 (0.2)
Have you ever breast fed			
Yes	81,667 (97.8)	33,851 (97.4)	37,800 (97.0)
No	349 (0.4)	277 (0.8)	297 (0.8)
Not applicable	1,366 (1.6)	596 (1.7)	884 (2.3)
Missing	90 (0.1)	41 (0.1)	01
Duration of breast feeding			
Up to 12 months	9,657 (11.8)	4,252 (12.6)	5,371 (14.2)
> = 12 months	71,438 (87.5)	29,830 (88.1)	32,295 (85.4)
Missing	572 (0.7)	231 (0.7)	134 (0.4)
Using oral contraceptive pill			
Yes	213 (0.3)	240 (0.7)	347 (0.9)
No	83,148 (99.6)	34,460 (99.1)	38,617 (99.1)
No response	117 (0.1)	65 (0.2)	18

Table 3 reveals the effect of training intervention on the knowledge of health care providers about breast cancer. We organized capacity-building workshops on breast cancer awareness at the block level for Medical Officers (MOs), Auxiliary Nurse Midwives (ANMs)/Lady Health Visitors (LHVs) and Accredited Social Health Activists (ASHAs). According to the existing manpower, 46 MOs out of 62, 401 ANMs/LHVs out of 527, and 377 ASHAs out of 1,046 attended the workshops. The remaining ANMs, ASHAs, Anganwadi workers, and Anganwadi helpers were trained in breast cancer awareness during the intervention activities for their respective community women. The level of knowledge improved in each parameter among the medical officers, ANMs/LHVs, and ASHAs after the intervention. It was observed that knowledge of medical officers about low-cost techniques for early detection as well as best treatment options for breast cancer increased from 83 to 100% and 72 to 86%, respectively. Also, knowledge of ANMs/LHVs regarding the most cost-effective method to detect breast cancer increased from 66 to 91%.

**Table 3:** Knowledge about breast cancer among health care providers in the selected districts.

Knowledge among Medical officers	Pre intervention (*n* = 46) Correct responseNumber (%)	Post intervention (*n* = 22) Correct responseNumber (%)
What is the most common cancer among woman in India? (Breast cancer)	25 (54.3)	20 (90.9)
Identify the most common risk factor for breast cancer (Nulliparous women)	29 (63.0)	17 (77.3)
Following is not a symptom of breast cancer? (Unusual bleeding)	24 (52.2)	14 (63.6)
Low-cost technique for early detection of breast cancer? (Breast self-examination)	38 (82.6)	22 (100.0)
Correct age group for clinical breast examination? (30–65 years of age)	3 (6.5)	4 (18.2)
When should the breast self-examination Done? (5–10 days post menstruation)	20 (43.5)	13 (59.1)
What are the best treatment options for breast cancer?	33 (71.7)	19 (86.4)
Knowledge among ANMs/LHVs	Pre intervention (*n* = 424) Correct response Number (%)	Post intervention (*n* = 287) Correct response Number (%)
What is the most common cancer among woman in India? (Breast Cancer)	340 (80.2)	277 (96.5)
Following is not a symptom of breast cancer? (Unusual bleeding)	225 (53.1)	170 (59.2)
Identify the most common risk factor? (Woman had never given child birth)	114 (26.9)	157 (54.7)
What is the most cost-effective method to detect Breast Cancer: BSE	279 (65.8)	260 (90.6)
Correct age for clinical breast examination (30–65 yrs. and once in 5 yrs)	52 (12.3)	97 (33.8)
Is community participation important in early detection of breast cancer	363 (85.6)	270 (94.1)
When should breast self-examination done? (5–10 days post)	159 (37.5)	182 (63.4)
Knowledge among ASHAs	Pre intervention (*n* = 322) Correct responseNumber (%)	Post intervention (*n* = 303) Correct responseNumber (%)
Knowledge about breast cancer symptoms?		
Poor (1–3)	96 (29.8)	59 (19.5)
Middle (4–6)	16 (4.97)	07 (2.3)
High (>6)	147 (45.7)	193 (63.9)
Knowledge about risk factors of breast cancer?		
Poor (1–3)	161 (50.0)	88 (29.1)
Middle (4–6)	27 (8.4)	30 (9.9)
High (>6)	71 (22.0)	141 (46.7)
Have you heard about breast self-examination?		
Yes	259 (80.4)	260 (85.8)
No	63 (19.6)	43 (14.2)

Similarly, knowledge among ASHAs about breast cancer symptoms and risk factors increased from 46 to 64% and 22 to 47%, respectively.

**Figure 1:**
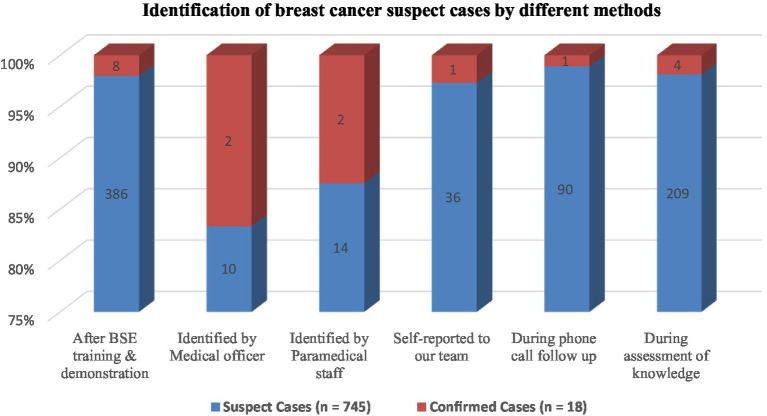
Identification of breast cancer suspect cases by different methods.

Figure 1 shows that a total of 745 suspect cases and 16 confirmed cases of breast cancer were identified. Most of the suspect cases (386, 51.7%) were identified after BSE training and demonstration, 28% identified during assessment of knowledge and 12% during phone call follow- up. When suspect cases were asked about symptoms of breast cancer, a majority (332, 44.6%) of them reported that they had a painless lump in their breast, 211 (28.3%) had a painful lump in the breast, 71 (9.5%) had painful lump in the armpit, 40 (5.4%) had change in size and shape of the breast, and 38 (5.1%) had reported unusual nipple discharge.

As revealed in Table 4, a total of 104,792 women were contacted during the first phone call follow-up for BSE compliance whereas 71,961 women were contacted during the second phone call follow-up. In both the follow-ups, it was observed that more than two third of women were practicing BSE, and about 40% educated other women about BSE. In total, 62 and 28 suspect cases were identified during the 1st and 2nd follow up, respectively.

**Table 4:** Phone call follow up status and bse compliance.

Follow up	District	Total call	Call attended	Talked to women	BSE compliance	Suspect cases	Educated others
1st	Jodhpur	57,097	35,400 (62.0)	21,619 (61.1)	14,951 (69.2)	13 (0.1)	8,862 (41.0)
1st	Jalore	13,331	8,449 (63.4)	5,574 (65.9)	3,706 (66.5)	27 (0.7)	2048 (36.7)
1st	Pali	34,364	21,453 (62.4)	13,587 (63.3)	9,532 (70.2)	15 (0.2)	5,501 (40.5)
Total		104,792	65,302 (62.3)	40,708 (62.4)	28,189 (69.1)	62 (0.2)	16,411 (40.1)
2nd	Jodhpur	35,726	18,974 (53.1)	10,694 (56.4)	6,919 (64.7)	11 (0.2)	4,873 (45.6)
2nd	Jalore	7,825	4,646 (59.4)	3,127 (67.3)	2,298 (73.5)	8 (0.3)	1,161 (37.1)
2nd	Pali	28,410	16,641 (58.6)	10,107 (60.7)	6,704 (66.3)	9 (0.1)	3,392 (33.6)
Total		71,961	40,261 (55.9)	23,928 (59.4)	15,921 (66.5)	28 (0.2)	9,426 (39.4)

**Figure 2:**
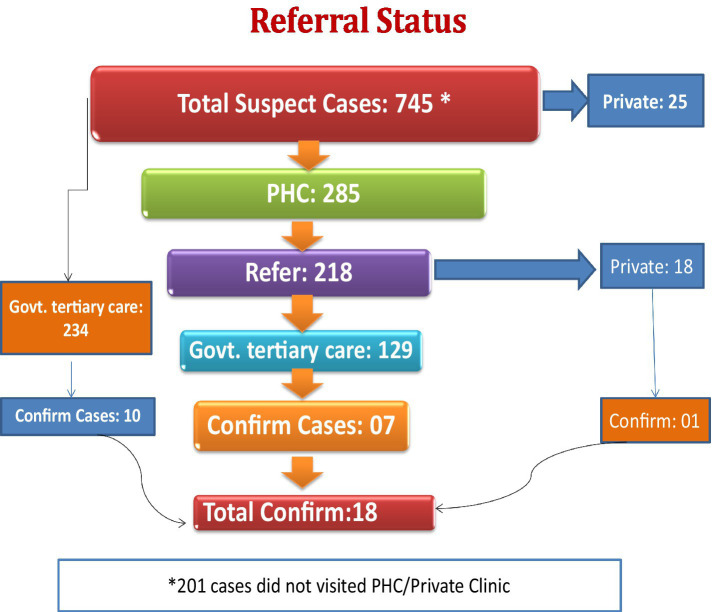
Referral status of breast cancer suspect cases.

All the suspect cases of breast cancer were referred to a nearby hospital/health center (Figure 2). Among them, 285 suspect cases visited their respective primary health centers, 234 went to Mathura Das Mathur hospital/All India Institute of Medical Sciences (tertiary health care hospitals) and 25 visited other private hospitals/clinics while 201 did not visit any health center/hospital/clinic. Out of 285 women who visited PHCs, the medical officers confirmed that 218 women had symptoms of breast cancer and referred them to MDM/AIIMS for further investigation and treatment. Out of these, 129 visited MDM/AIIMS, Jodhpur/Bangur hospital, Pali and rest of them visited other tertiary care hospitals/private hospital as per their convenience. Overall, 18 women were diagnosed to have breast cancer.

After the identification of suspect cases, many of them (201/745) did not visit PHC for further check-up, even though we tried to persuade them through phone calls and, home visits, and offered them assistance and transport facilities for check-ups. The reasons given for non-compliance were not understanding the significance of clinical examination, not taking it seriously, treatment-related anxiety, time constraints, financial hardships, family obligations, lack of belief in the health care delivery system, etc.

## Discussion

In this study, an attempt was made to test the feasibility of using BSE as a method of improving early detection of BC thereby strengthening breast cancer screening program in three districts (Jodhpur, Jalore, Pali) of Rajasthan and develop a referral mechanism for diagnosis and treatment of suspect cases at tertiary health care settings.

Formative phase data shows that around 0.2 percent of women were performing breast self-examinations (BSE). However, phone call follow-ups showed that community women were performing BSE and raising awareness about BSE among other women. This approach proved feasible because it required no additional personnel or resources. Any woman can perform a BSE during bathing or before sleeping.

To achieve this, first, we assessed the knowledge of all the women (aged 30–65 years) of these districts regarding breast cancer, educated them about breast cancer, and trained them about the practice of breast self-examination using a printed booklet and demonstrating on dummy breast model. We also assessed the knowledge of the medical officers, ANMs/LHVs, and ASHAs of selected blocks in these districts about different aspects of breast cancer and trained them for identifying suspect cases. After the identification of suspect cases, a mechanism was developed to refer these cases to nearby hospitals/health centers for further follow-up. We observed that strengthening breast cancer screening program using strategic education and awareness program is feasible and can be sustained if the project is converted from project mode to program mode and executed by the state government and central government.

We faced certain challenges in carrying out this project and appropriate actions were taken accordingly. First, women were reluctant for the survey in the absence of paramedical staff/AWW/AWH. Thus, we took the assistance of ANM/ASHA/AWW/AWH and community volunteers before carrying out various activities. Second, health care providers asked for official order to assist in project activities. In response to that, request letters were sent to Chief Medical Health Officer (CMHO) and Director, ICDS to issue official orders for health care providers to cooperate in the project activities. Third, all the study participants could not be covered which might be due to one-time visit to a household as per protocol and participants’ unavailability during the visit. Thus, to cover more study participants; the message was conveyed through ASHA/AWW/AWH of respective areas the day before assessment and BSE training. Fourth, suspect cases of breast cancer requested transport support to visit PHC/CHC/tertiary care center. MO/ANM/ASHA and research staff of concerned areas took the responsibility to help the suspect cases visit nearby PHC/CHC/tertiary care centre. Also, capacity building of all medical and paramedical staff could not be done due to their engagement in other health care activities. Moreover, due to the COVID-19 pandemic, the study implementation plan could not be timely executed.

However, in spite of these challenges, we were able to assess the knowledge of 157,225 women in three districts (Jodhpur, Jalore, Pali) representing a large percentage (40%) of the population of these districts. We made 218,978 women aware by using printed booklets and dummy breast models of the disease and trained them on how to practice breast self-examination. The importance of BSE among women for early detection of breast cancer is evident as more than half of the suspect cases were identified after BSE training and demonstration. We trained 824 medical officers/ANMs/AWWs/ASHAs for correctly identifying suspect cases and referring them to appropriate healthcare facilities. The capacity-building workshops proved effective in increasing the knowledge of medical and paramedical personnel for early detection of breast cancer.

The, intervention implemented was effective, as evidenced by the phone call follow-up for BSE compliance and the suspect case reporting process, which proved its effectiveness. We could not obtain documents from all confirmed cases, even though most were detected in the first stage of breast cancer.

Based on the study results, it can be said that the entire study process was beneficial for both healthcare professionals and the community women. Now, women have learned about the signs, symptoms, and BSE, and they are educating and sharing their experiences with other women. This will be helpful for the early detection of breast cancer and timely treatment, which can save lives and reduce the disease burden. This program also helps dispel myths and reduce women’s reluctance about the disease.

### Limitations

This study was limited to three districts of Rajasthan state and there were certain operational difficulties as mentioned in the study. Additionally, recall bias might affect responses relating to BC knowledge of signs, symptoms, and risk factors since they depend on the participants’ understanding abilities.

### Recommendations

The project was designed jointly with the Government of Rajasthan. In the project, it has been demonstrated in three districts (Jalore, Pali, and Jodhpur) that the objectives are achievable and can be sustained if the project is converted from project mode to programme mode and executed by the state government. Therefore, it is recommended that:

- Breast self-examination (BSE) is a crucial tool for increasing breast cancer awareness since it helps women get familiar with their breasts, notice and feel any deviations from the usual, and quickly report any changes they notice.
- BSE may decrease the barriers preventing women from undergoing clinical breast examination and mammography.
- Effective educational interventions are needed to encourage women to maintain frequent breast cancer awareness and breast self-examination.
- In order to detect breast cancer early, all medical and paramedical personnel of the state should be trained.
- At the grass root level ANMs may be instructed to train women (aged 30–65 years) regarding breast self-examination and may follow-up for breast self-examination compliance annually by the ASHAs.
- There is need to enhance ultrasound (for breasts) facilities at community health centre and district hospital.
- There is need to upgrade facilities like FNAC, Mammogram and Biopsy at district hospital.
- It was observed that patients and their families experience significant hardships as a result of late-stage BC.

Therefore, considering the above recommendations, we recommend approaching the government of Rajasthan to adopt the project and transition it into a program. Nevertheless, we hope that the lessons learned through our efforts in this project will assist in its replication, either in part or in full, in other parts of India or in other developing countries.

## Data availability statement

The original contributions presented in the study are included in the article/supplementary material, further inquiries can be directed to the corresponding author.

## Ethics statement

The studies involving humans were approved by National Institute for Implementation Research on Non-Communicable Diseases, Jodhpur. The studies were conducted in accordance with the local legislation and institutional requirements. Written informed consent for participation in this study was provided by the participants' legal guardians/next of kin. The manuscript presents research on animals that do not require ethical approval for their study.

## Author contributions

RS: Conceptualization, Formal analysis, Funding acquisition, Investigation, Methodology, Project administration, Supervision, Visualization, Writing – review & editing, Data curation, Resources, Validation.

RH: Data curation, Formal analysis, Project administration, Software, Validation, Writing – review & editing, Conceptualization, Methodology, Supervision. AP: Writing – original draft, Writing – review & editing. GT: Funding acquisition, Methodology, Project administration, Supervision, Writing – review & editing. AS: Writing – review & editing, Data curation, Formal analysis. MT: Data curation, Investigation, Writing – review & editing. PK: Data curation, Formal analysis, Investigation, Project administration, Writing – review & editing.
